# Dynamics of Forest Fragmentation and Connectivity Using Particle and Fractal Analysis

**DOI:** 10.1038/s41598-019-48277-z

**Published:** 2019-08-22

**Authors:** Ion Andronache, Marian Marin, Rico Fischer, Helmut Ahammer, Marko Radulovic, Ana-Maria Ciobotaru, Herbert F. Jelinek, Antonio Di Ieva, Radu-Daniel Pintilii, Cristian-Constantin Drăghici, Grigore Vasile Herman, Alexandru-Sabin Nicula, Adrian-Gabriel Simion, Ioan-Vlad Loghin, Daniel-Constantin Diaconu, Daniel Peptenatu

**Affiliations:** 1 Research Center for Integrated Analysis and Territorial Management, University of Bucharest, Bucharest, 030018 Romania; 2 Research Institute of the University of Bucharest, Bucharest, 050107 Romania; 3 Helmholtz Centre for Environmental Research - UFZ, Leipzig, 04318 Germany; 4 GSRC, Computational Medicine Lab, Medical University of Graz, Graz, 8010 Austria; 5 Laboratory of Cancer Cell Biology, Institute for Oncology and Radiology, Belgrade, 11000 Serbia; 6 Faculty of Geography, University of Bucharest, Bucharest, 010041 Romania; 7 Centre for Research in Complex Systems, Charles Sturt University, Albury, Australia; 8 School of Community Health, Charles Sturt University, Albury, Australia; 9 Macquarie University, Faculty of Medicine & Health Science, Department of Clinical Medicine, Sydney, NSW Australia; 10 Department of Geography, Tourism and Territorial Planning, Faculty of Geography, Tourism and Sport, University of Oradea, University Street, no. 1, Oradea, Romania; 11 Centre for Research on Settlements and Urbanism, Faculty of Geography, Babeş-Bolyai University, Cluj-Napoca, 400006 Romania; 12 National Institute for Economic Research Costin C. Kiriţescu, Romanian Academy, 050711 Bucharest, Romania; 13 Faculty of Geography, Babeş-Bolyai University, Cluj-Napoca, 400006 Romania

**Keywords:** Environmental impact, Environmental impact

## Abstract

The ever decreasing area of forests has lead to environmental and economical challenges and has brought with it a renewed interest in developing methodologies that quantify the extent of deforestation and reforestation. In this study we analyzed the deforested areas of the Apuseni Mountains, which has been under economic pressure in recent years and resulted in widespread deforestation as a means of income. Deforested surface dynamics modeling was based on images contained in the Global Forest Database, provided by the Department of Geographical Sciences at Maryland University between 2000 and 2014. The results of the image particle analysis and modelling were based on Total Area (ha), Count of patches and Average Size whereas deforested area distribution was based on the Local Connected Fractal Dimension, Fractal Fragmentation Index and Tug-of-War Lacunarity as indicators of forest fragmentation or heterogeneity. The major findings of the study indicated a reduction of the tree cover area by 3.8%, an increase in fragmentation of 17.7% and an increase in heterogeneity by 29%, while fractal connectivity decreased only by 0.1%. The fractal and particle analysis showed a clustering of forest loss areas with an average increase from 1.1 to 3.0 ha per loss site per year. In conclusion, the fractal and particle analysis provide a relevant methodological framework to further our understanding of the spatial effects of economic pressure on forestry.

## Introduction

In view of its effect on climate, deforestation is athe threatening challenge for contemporary society. The major factors leading to the decline of forested areas include an expansion of agricultural land^1–4^, human settlements^5^, timber demand^6^, mining exploitations and population growth^7^ leading to environmental and economic challenges in the long term. Therefore the aim of the local or regional authorities is to reduce deforestation and to regenerate the existing forests wherever possible due to their importance in supporting the biotic system, regional and global climate change, the preservation of atmospheric oxygen and carbon, conservation of biodiversity and benefits to local communities^6,8–12^.

Forest fragmentation is a major result of deforestation^13,14^. It leads to habitat modification^15^, and subdivision of plant and animal populations. Thus, changes in species interactions occur^13,14^, leading to further tree mortality and destruction as observed at the edges of forest fragments^16^. Previous studies have suggested that forest fragmentation may have profound effects on biodiversity^17,18^ and differ among plant species^19^ resulting in some plant species having lower survival rate^20^. Forest fragmentation also generates a decline in forest bird populations^21^ by reducing the nesting success^22,23^ and the diversity of mammalian species^24^. In addition, forest fragmentation generates microclimatic changes^14^ with the risk of extinction of thousands of species^25,26^ associated with a lack of food, shelter and increased risk of attacks by carnivorous mammals^27^. An analysis of forest cover also indicates large carbon emissions from fragmented forests due to higher tree mortality at forest edges^28^. However; fragmentation may exert positive effects on increasing the abundance of the lianas if the severity of forest fragmentation intensifies^29^. Research on the impact of deforestation has revealed the complexity of this process, determined by the size of the deforested areas and their fragmentation patterns. that can impact the environment in diverse ways including frequency of flooding depending on the degree and type of logging^30^.

A forest fragmentation model based on the principles of percolation theory has been previously used for evaluation of the state of fragmentation. Results indicate that forest fragmentation is close to the critical point of percolation, which means that the number of small forest fragments will expand exponentially with increasing deforestation^31^.

Additional measures describing the state of forest fragmentation such as the rank occupancy-abundance profile^19^, the relation between forest patch size and proximity of forest to non-forest edge^26^, mean patch size, patch density and edge density provide further evidence of the impact of deforestation^32^.

Remote sensing has largely improved analysis of forest exploitation through logging and its impact on forests by changes in satellite, airplanes or UAV’s images. Forests are currently monitored least annually by satellite images globally as well as at regionally or locally^33^.

The complexity of the deforestation phenomenon calls for new analystical approaches. The proposed methodology in this study complements the approaches that have proven useful in previous research by applying Local Connected Fractal Dimension (*LCFD*), Tug-of War Lacunarity *(Λ*_*T-o-W*_) and Fractal Fragmentation Index (*FFI*)^34–39^.

## Results

Particle analysis was carried out alongside fractal analysis in an attempt to tangibly describe what a highly abstract fractal analysis actually measures. The aggregated results can be found in the Supplementary Material as Table S1.

### Particle analysis of the forested, deforested (loss) and regenerated area dynamics

**Figure 1:**
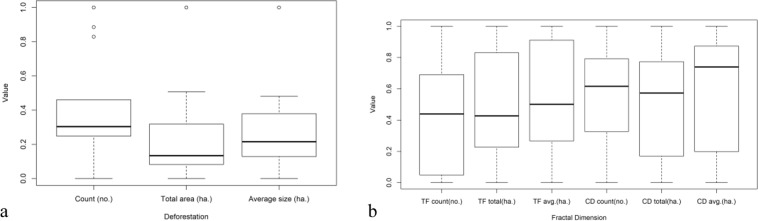
The dynamics of tree cover areas (ha.), particle count and average tree cover size at Apuseni Mountains, between 2001–2014 using standardized values. Forest loss areas (**a**), and tree cover (**b**) represent yearly data; cumulative forest loss areas (in black) represent cumulative forest loss areas (2001, 2001–2002, 2001–2003, 2001–2014). Gain areas are not presented because only one image was analyzed: cumulative gain areas 2001–2014. (TF means tree cover and CD means cumulative forest loss areas).

Figure 1a,b present the dynamics of the Apuseni Mountain tree cover and forest loss areas. The tree cover area has decreased by 3.8%from a total of 794,005 ha in 2000 to 764,002 ha in 2014. The spatial dynamics of forested, loss and gain areas was determined using Particle Analysis data (Table S1).

**Figure 2:**
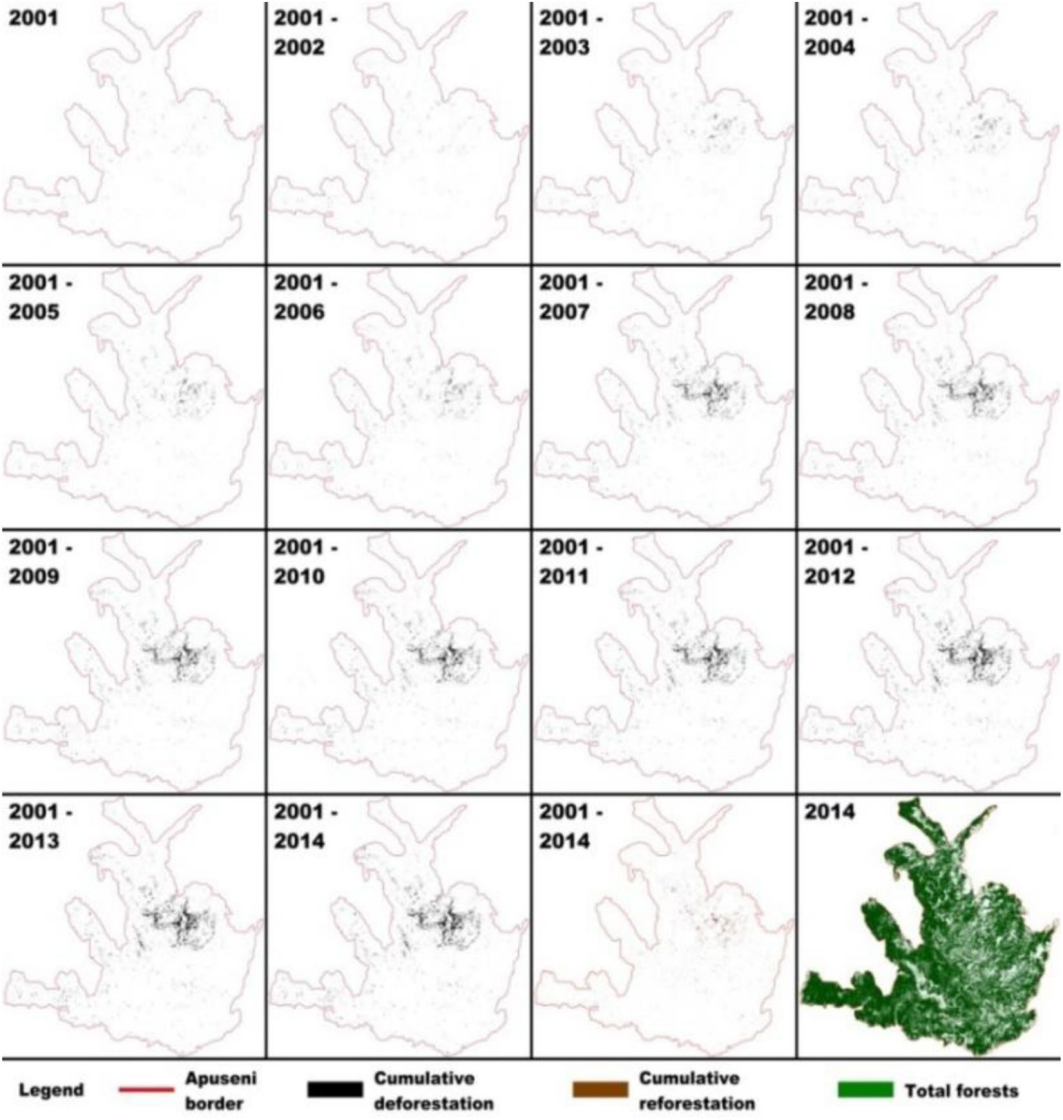
Dynamics of cumulative forest loss areas (Cumulative deforestation), in relation to cumulative gain areas (Cumulative reforestation) and tree cover (Total forests) in Apuseni Mountains between 2001 and 2014. From QGIS Development Team (2018), QGIS Geographic Information System. Open Source Geospatial Foundation Project. http://qgis.osgeo.org.

**Figure 3:**
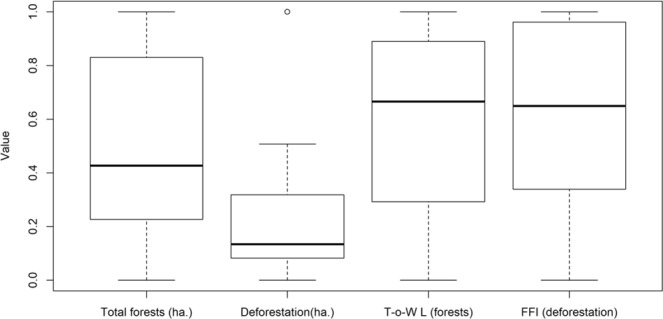
The local effects of heterogeneity of the forest loss areas (deforestation) on the tree cover (forests) measured by *Λ*_*T-o-W*_ (*T-o-W L*) compared with tree cover area (ha.), forest loss areas (ha.) and forest fragmentation measured by *FFI* (violet), using standardized values. Spearman’s correlation coefficients are shown in Table S2 in Supplementary Material.

Until 2004, forest loss areas were dispersed in small patches. However since then a clustering process occurred through a cluster development in the Gilău, Muntele Mare and Vlădeasa Mountains (Fig. 2). This coincided with a more pronounced scatter of total forest and thus led to a higher fragility of tree cover areas. Our measures have shown that, between 2000 and 2014, only 46.6% of the total forest loss areas have been regenerated (Table S1, Fig. 3).

By a particle analysis *particle count* parameter, we showed that out of 19,771 ha of forest loss areas, 9,951 clusters were formed while the rest remained as isolated forest loss areas. As a result of forest loss areas, the tree cover has become more fragmented, with the appearance of 727 new independent forest loss areas which amounted to 3.7% of cumulative forest loss areas.

Interesting results were also obtained by the *average size* analysis of forest loss areas. In periods with intense logging the *average size* of the loss sites exceeded 1.5 ha but in years with less logging activity the *average size* was less than 1.0 ha per loss site (Fig. 1a). This has revealed a clustering process: the average forest loss areas increased from 1.1 to 3.0 ha per loss site, over the study period. The highest increases in forest loss areas occurred in 2007 and 2012, years with the largest forest loss areas. Forest fragmentation also led to a decrease from 99.6 to 87.8 ha per average tree cover site (Table S1). The most intense fragmentation occurred in periods with the largest forest loss areas: 2009–2010 and 2012 (Fig. 1a).

### Fractal analysis

Local Connected Fractal Dimension (*LCFD*) was employed for the first time in forest analysis to measure the degree of connectivity described as a connection of each forest pixel with eight neighboring forest pixels from satellite images, previous exploited only in biology and medicine^40^.

*LCFD* analysis of forest loss areas indicated that the greatest connectivity was during years with the largest forest loss areas, because the deforestation made in several patches. The least connectivity of forest loss areas, was found in years with very small forest loss areas. The highest increase of connectivity was recorded when forest loss areas were moderate, below average, but homogeneously organized and compact. As the cumulative loss area increased, its connectivity decreased. This finding suggested that forest loss areas did not have a strong influence on the spatial treecover complexity in the Apuseni Mountains, because were done in small patches. (Fig. 2), though this decrease was only 0.1%. *LCFD* actually reflected the patch sizes, because of the strong correlation (0.95–1.00) between *LCFD* with tree cover and cumulative forest loss areas size (Fig. 2). Between 2001 and 2014 *LCFD*s of regenerated areas were lower by 51% compared to *LCFD*s of forest loss areas. This may be due to a regeneration occurring in smaller and highly spatially fragmented areas, leading to a lower connectivity between the forest patches (Table S1). Using LCFD analysis has revealed that forest loss areas increase has little effect on the tree cover fractal connectivity and may therefore contribute substantially to fragmentation.

The *FFI* index offers information regarding the fragmentation or the degree of compaction of an object. For the analyzed period, the forest areas decreased by 3.8% with a *FFI* decrease of 17%, thus indicating an increase in fragmentation of the tree cover (Table S1).

The highest decrease in *FFI* for the tree cover areas was registered in 2001 and 2007 when forest loss areas were characterized by low-fragmentation and 68% of forest loss areas occurring in new locations. The smallest decrease in tree cover *FFI* was in 2002 when forest loss areas distribution occurred mainly in small forest patches (Table S1).

Further minimal fragmentation occurred in 2007 and 2011, but fragmented loss areas of 34–39% were registered in 2013–2014 (Fig. 3). The higher the loss surface, the more compact it was. Thus, the years with intense forest loss areas were also associated with their increased compactness.

The *FFI* of regenerated areas between 2001 and 2014 was 51% lower compared to *FFI* of the deforestation areas (Table S1).

Interesting results were obtained by the Tug-of-War Lacunarity (*Λ*_*T-o-W*_) analysis. It revealed maximal heterogeneity in years with dominant forest loss areas in new, and relatively large areas (Table S1). In contrast, the maximum homogeneity of the spatial forest loss areas distribution occurred when forest loss areas occurred as continuation of loss patches and in relatively small areas. *Λ*_*T-o-W*_ of the tree cover did not show a continuous downward trend. Because the deforestation was heterogeneous, the value of *Λ*_*T-o-W*_ increased by 29%, even though the forested areas decreased by only 3.8%. This fractal parameter therefore provided independent information as it showed low correlation with the standard forest parameters such as tree cover and forest loss areas (Table 2 Supplementary Material).

## Discussion

The increasing fragmentation observed during the study period was most likely due to legislative changes in the management of the forests, the most important being the retrocession of large areas and the fast logging, as well as illegal logging. Fragmentation of forests is a stage in the loss of compact forest areas. The way forests are fragmented provides information on how to intervene in their exploitation. Illegal exploitation of wood masses is manifested by large fragmentation, and legal logging through compact cuts. Analysis of fragmentation over extended periods of time helps to forecast an evolution of economic pressure on forest resources globally.

This study measured the dynamics of forest loss areas by use of fractal and particle analysis features for a large region in Romania.

The results indicated that a tree cover decreased every year of the study while fragmentation increased. Such continuous decrease in tree cover was due to increased legal and illegal deforestation in the period of economic and legislative changes that encouraged logging. (Fig. S1, in Supplementary Material).Furthermore, forest loss areas have occurred in “jumps” corresponding to a transfer of significant portions of forested land from state property to the former owners prior to nationalization in 1948 (Fig. 1a).

The fractal indicators complement each other by providing different information. Connectivity information obtained by *LCFD* is supplemented with fragmentation/compaction (*FFI*) and heterogeneity/homogeneity (*Λ*_*T-o-W*_). This complementary analysis has allowed us to highlight how the loss in forest areas occurred and how it spatially affects the tree cover of the Apuseni Mountains.

*LCFD* quantified the local effects of forest loss areas on fragmentation of the forests through analyzing changes in connectivity of the forest. *LCFD* analysis of deforestation indicated that the largest connectivity occurred in years when large fragmented and heterogeneous deforestation alternated with homogenous forest loss areas. The lowest connectivity was registered during the years with very small fragmented but homogenous forest loss areas. This occurred by the merging of tree clusters in forest loss areas. *LCFD* analyses of forest loss areas showed that connectivity was directly proportional to the expansion of forest loss areas and also to the degree of homogeneity of the tree cover area clustering.

We have shown that *LCFD* might also be useful in assessing the local variations in complexity for forest loss areas or regeneration, unlike the global fractal dimension approach used in previous studies.

*FFI* analysis was used for determining the extent of forest fragmentation. Dynamics of *FFI* showed that the trend in forest loss areas clustering is based on their spatial extensions, especially after 2005 and that forest loss areas were less influenced by fragmentation compared to regeneration. Moreover, in this study we provide a substantial improvement in *FFI*’s ability to quantify fragmentation by correlating the results for the first time with fractal connectivity and particle analysis of binary images.

We have already reported^35^ that mountainous, well-forested counties have a low degree of forest fragmentation, with the *FFI of* 0.13, which was 0.08 higher compared to hilly counties with less forest.

Another recent study^6^ has shown a high degree of fragmentation in 2014 of forests in Maramures County with *FFI* of 0.09. Draghici *et al*. (2017) reported a lower degree of fragmentation for the Northern Carpathian Mountains of Romania, with *FFI* of 0.11 in 2014^36^ compared to the current results. Higher *FFI* values for the Apuseni Mountains of 0.15 in 2014 were found in the current study. This indicates that the forests of the Apuseni Mountains compared to the Northern Carpathian Mountains of Romania or the Maramures and Suceava counties still have a low degree of fragmentation despite the widespread emergence of forest loss areas in the period between 2000–2014. The results are clearly different due to the different patterns of deforestation, the greater severity of deforestation in the northern counties of Maramures and Suceava, together with the presence of several protected areas in the Apuseni Mountains, which are very compact.

Previous studies have shown that the largest increase of forest fragmentation occurred during the most aggressive loss area expansion in Romania: in Suceava County *FFI* was 0.06, followed by 0.04 in the Northern Carpathian Mountains and 0.021 in Maramures County^6^. With the same reference range and use of new images, we now report a *FFI* reduction of 0.03 calculated for the Apuseni Mountains, comparable to that for Maramures County. This was due to only 3.8% of the tree cover being lost between 2000 to 2014. Previously, it was reported that for the same period Maramures County lost 5.06% of the tree cover^6^, the Nordic Group 6.75%, while Suceava County lost 9.5%^36^.

*Λ*_*T-o-W*_ analysis offered the best perspective on the effect of heterogeneous logging on tree cover areas. This pattern of forest loss areas was most likely a result of the legislative changes, but also influenced by the type of ownership of the forest and the ability of the landlord to deforest or pay for deforestation. Thus, the lowest values of the *Λ*_*T-o-W*_ were occurring in the years with intensive expansion of forest loss areas and forest fragmentation, indicating clustering, with the appearance of new small forest fragments detached from the compact forest areas. This compares to fewer new fragments that appeared in periods of low loss.

Our study has some limitations that must be addressed. The images used, with a spatial resolution of 30 m, allowed us to capture only a coarser picture of forest patterns. The use of more detailed images would resolve this restriction^41^ and thus improve *FFI* or *LCFD* accuracies. However, the 30 m resolution used is the state-of-the-art and currently the best resolution. Future advances in satellite imagery are expected to improve this situation. Another limitation of *FFI* analysis is that it is computed for the whole image without giving information regarding the regional variations. A scaling limitation is another general problem in image analysis, with single pixels as the smallest scale and the size of the entire image as the largest scale. Furthermore, natural objects are also intrinsically limited in scaling due to the finite dimension of their structural units^37^.

In the current study all the images of forested, loss and gain areas were on the same scale. This is very important for fractal analysis because the algorithms used are extremely sensitive even for the smallest variation in the size and shape of an image^38^. It is necessary to clarify that, contrary to ideal fractals, natural patterns can be considered fractals only for limited scale intervals. This is due to the finite dimensions of the elements, in this case the tree groups, which form the whole forest.

Taken together, we conclude that a combination of particle and fractal analysis can be of use in assessing forest fragmentation as these analyses provide different and compatible information. The results obtained show that the analysis of changes in the distribution and fragmentation of forestsby use offractal fragmentation, fractal connectivity and lacunarity, along with particle analysis, provides significant novel insights into the understanding of how economic pressure on forest resources. Fragmentation of the forest is also relevant for complex flood risk calculation methodologies, with many studies that emphasize the need to introduce fragmentation into classical models^34,35,39,41^.

## Methods

### Study area

**Figure 4:**
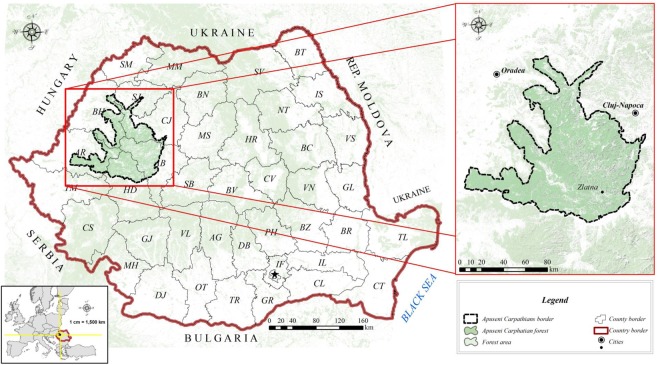
Geographical study area of Apuseni Mountains (QGIS Development Team (2018). QGIS Geographic Information System. Open Source Geospatial Foundation Project. http://qgis.osgeo.org).

The Apuseni Mountains were chosen as a case study because this mountain group is characterized by strong fragmentation of the landforms and subjected to increasingly intense deforestation. The Apuseni Mountains are part of the Western Carpathians and are bounded by the Mureşului valley to the south and the Someşului and Barcăului valleys to the north (Fig. 4). With a diversity of landscapes imposed by the petrographic mosaic, this mountain area is morphologically divided into the Bihor Mountain group including Bihor, Vlădeasa, Gilău and Muntele Mare Mountains; the Zarand Mountains, the Codru-Moma Mountains, the Pădurea Craiului Mountains, the Metaliferi Mountains, the Trascău Mountains and the Culmea Şesului Mountains. Geographically the extended area is located at 21°593704N, 45°866423E to 23°716872N, 47°220550E and administratively, the case study area is located at the junction North-West, Central and West development regions, with six counties Bihor, Sălaj, Cluj, Alba, Hunedoara, and Arad. The dominant species in these forests are Pinus mugo, Picea abies, Abies alba, Quercus robur, and Fraxinusexcelsior^42^. These species are native to the two most deforested counties of Romania: Cluj and Alba, three counties with moderate deforestation: Bihor, Hunedoara and Arad, and one with less significant deforestation being Sălaj. Within their morphological limit, the Apuseni Mountains have a total surface area of 11,645 km^2^ and a perimeter of 1000 km^43^.

### Image processing

Fractal analysis is able to detect changes on satellite images deriving from vegetation evolution. Image processing was based on the Global Forest Database, provided by the Department of Geographical Sciences, Maryland University. This database is the result of 654,178 Lands at 7 ETM+ images taken between 2000 and 2014^33^. Follows: an image of Tree canopy cover for the year 2000 which is the starting point for this analysis, that represent the years of cumulative forest cover loss event from 2001 to 2014 compared to year 2000 and one image of year of cumulative forest cover loss event. For the gain areas only cumulative regeneration from 2001 to 2014 for each year was analyzed^33^.

The three images were post processed by GIS methods (reprojecting the images from WGS84 to Stereo70, the national coordinate system, to obtain metric results) by extracting a subset of the study area from each image and computing the loss area in meters for each year between 2000 and 2014. The pixel resolution for each image was about 30 m. Both Figs 2 and 4 were firstly generated with QuantumGIS (Version 2.2.0 available at http://download.osgeo.org/qgis/windows/). For image stacking in Fig. 2 was used ImageJ Fractal software, and for cartographic layout in Fig. 4 Inkscape (0.48.2) was used after the main images were made in QuantumGIS. The Administrative boundaries were downloaded from the Romanian Authority which reports data to Eurostat (European Statistical Office) from which both national boundaries and European countries border were downloaded.

GIS methods were used to extract vector data from the metric projected images in GeoTIFF format with 30 m resolution from the Global Forest Change 2000–2014 database and to create grayscale TIFF images. The images were produced by keeping the same scale, orientation, chromatic and exporting resolution from the vector data classified for each loss year. Keeping the mentioned parameters, the fractal analysis is more precise. The 14 TIFF images exported from the initial data were automatically binarized using ImageJ 1.51p software (Wayne Rasband, National Institute of Health, USA, 1997)^44,45^.

### Particle analysis

The loss and gain areas and their impact on the dynamics of forest fragmentation were investigated by particle analysis including particle count, size, and average size, where a particle refers to a forest patch. Gain areas were also evaluated. Particle counter plug-in^46^ of ImageJ was used for this analysis^47^.

### Fractal analysis

Because the forests analysed here are morphologically complex, showing very often fragmented and non-uniform patterns and because the particle descriptors depend on the scale of observation^48^, the forest images were further examined by fractal analysis as this is a scale-invariant method. Fractal dimension (FD) as the main fractal parameter predominantly describes the degree of morphological complexity^49^.

Fractal Fragmentation Index (*FFI*) measures the fragmentation/compaction of the forest patches. Tug-of-War Lacunarity (*Λ*_*T-o-W*_*)* was used to measure the degree of the heterogeneity, i.e. to investigate whether the forest patches are arranged chaotically or more regularly.

Local Connected Fractal Dimension (*LCFD*) is a fractal index of complexity, quantifying connectivity changes at varying scales. The relationship is expressed in (Eq. (1):

1 $$M({\epsilon })\propto F{{\epsilon }}^{LCFD}$$

and (Eq. (2)):

2 $${\rm{L}}CFD=\frac{\mathrm{log}\,[M({\epsilon })]}{\mathrm{log}\,({\epsilon })}$$

where F is a mass pre-factor, ${\rm{M}}\,(\epsilon )\,$is the number of locally connected pixels (a connection with eight neighbors) in a side-by-side box ε^50^.

The *LCFD* value equals 1.0 when the object is a one-dimensional straight line. *LCFD* equals 2.0 when the object is two-dimensional and completely covered^51^. Pixels in the 8 × 8 environment of the seed pixel are considered to be connected. This basic rule is applied to find the set connected for a certain predetermined, arbitrary distance around the seed pixel.

*LCFD* was computed using ImageJ software and FracLac 2016 Apr 120248 a 502 plugin^52^.

The Fractal Fragmentation Index (*FFI*) provides information on the degree of fragmentation using the Box-Counting algorithm. *FFI* can be interpreted as a compaction index (Eq. (3))^35^:

3 $$FFI\,={D}_{B-C}A-{D}_{B-C}P=\mathop{\mathrm{lim}}\limits_{\varepsilon \to 0}(\frac{\mathrm{log}\,N(\varepsilon )}{\mathrm{log}\,\frac{1}{\varepsilon }})-\mathop{\mathrm{lim}}\limits_{\varepsilon \to 0}(\frac{\mathrm{log}\,N^{\prime} \,(\varepsilon )\,}{\mathrm{log}\,\frac{1}{\varepsilon }})$$

where ${{\rm{D}}}_{{\rm{B}}-{\rm{C}}}{\rm{A}}\,$ is the fractal dimension of the summed-up areas, ${{\rm{D}}}_{{\rm{B}}-{\rm{C}}}{\rm{P}}$ is the fractal dimension of the summed-up perimeters, ε is the side length of the box, N (ε) is the number of non-overlapping and contiguous boxes required to cover the area of the fractal object being analyzed and N′ (ε) is the number of non-overlapping and contiguous boxes, necessary for covering the perimeter of the analyzed fractal object^35^. As the zero limits cannot be applied to digital images, DA and DP were estimated by the slope of a double logarithmic plot^53^.

According to a previous study of Andronache *et al*.^35^, *FFI* values close to 1.0 indicate compact objects, while *FFI* values approaching zero indicate very small and fragmented objects. *FFI* = 0 reflects a very small forest area, equal to the size of one pixel in an image with ${D}_{B-C}A={D}_{B-C}P$^35,39^.

*FFI* was calculated by using the *FFI* plugin^40^ for the IQM 3.5 software^54^.

While *FFI* quantifies how much compact or fragmented the space occupied by the forest is, the lacunarity quantifies how space is occupied. In order to assess the degree of heterogeneity of forest loss areas compared to gain areas and their effects on forest, the Tug-of-War lacunarities (*Λ*_*T-o-W*_)^55^ were used. *Λ*_*T-o-W*_ indicates mainly the manner of forest loss areas where increasing values indicate a chaotic distribution of the forest loss areas and vice versa. *Λ*_*T-o-W*_ was calculated based on the equation (Eq. (4)):

4 $${\Lambda }_{T-o-W}=\frac{N(r){Z}^{2}}{{L}^{2}}$$

with ${\rm{N}}({\rm{r}})\,$being the total number of boxes.

Z^2^ is the second moment for each width and is approximated by

5 $${Z}^{2}\approx \mathop{\sum }\limits_{i=1}^{N(r)}\,p{(r,i)}^{2}$$

With *p (r,i)* the number of occupied sites in the *i*-th box and finally, *L* is approximated by the mean of the occupied sites by

6 $${L}^{2}\approx {(\mathop{\sum }\limits_{i=1}^{N(r)}p(r,i))}^{2}$$

Actually, *Λ*_*T-o-W*_ was calculated by using the Fractal 2D Dimension plugin^56^ for the IQM 3.2 software.

For a better graphic representation we used standardized values because particle and fractal analyses have different units and implicitly different maximum and minimum values. Standardization according to Eq. (7) allowed us to present results comparably and conveniently in a graphical format.

7 $${\rm{Standard}}\,{\rm{value}}=\frac{{\rm{Vnom}}-{\rm{Vmin}}}{{\rm{Vmax}}-{\rm{Vmin}}}.$$

Where Vnom = nominal value, Vmax = maximum value, Vmin = minimum value

Standardized values are between 0–1.0.

Correlations were determined by computing the Spearman’s rank correlation coefficient.

## Supplementary information

Table 1Table 2
