# Supplementary material for: Dynamics of Forest Fragmentation and Connectivity Using Particle and Fractal Analysis
Source: Sci Rep. 2019 Aug 22;9:12228. doi: 10.1038/s41598-019-48277-z (PMC6706383; doi:10.1038/s41598-019-48277-z)
Supplement: Supplementary file 1 — Table 1Table 2 [file 41598_2019_48277_MOESM1_ESM.docx]

**Dynamics of Forest Fragmentation and Connectivity Using Particle and Fractal Analysis**

Ion Andronache^1,+^, Marian Marin^1,+^, Rico Fischer^3^, Helmut Ahammer^4^,
Marko Radulovic^5^, Ana-Maria Ciobotaru^1^, Herbert F. Jelinek^7,8^, Antonio Di Ieva^9^,
Radu-Daniel Pintilii^1,+^, Cristian-Constantin Drăghici^1,+^, Grigore Vasile Herman ^10,+^,
Alexandru-Sabin Nicula^11,12,13,+^, Adrian-Gabriel Simion^1,6^, Ioan-Vlad Loghin^1,2,6^, Daniel-Constantin Diaconu^1,+^and Daniel Peptenatu^1,+,^*

^1^Research Center for Integrated Analysis and Territorial Management, University of Bucharest, Bucharest 030018 Romania;

^2^ Research Institute of the University of Bucharest, Bucharest050107, Romania;

^3^ Helmholtz Centre for Environmental Research - UFZ, Leipzig 04318, Germany;

^4^ GSRC, Computational Medicine Lab, Medical University of Graz, Graz 8010, Austria;

^5^ Laboratory of Cancer Cell Biology, Institute for Oncology and Radiology, Belgrade 11000, Serbia;

^6^ Faculty of Geography, University of Bucharest, Bucharest 010041, Romania;

^7^ Centre for Research in Complex Systems, Charles Sturt University, Albury, Australia;

^8^ School of Community Health, Charles Sturt University, Albury, Australia;

^9^ Macquarie University, Faculty of Medicine & Health Science, Department of Clinical Medicine, Sydney, NSW, Australia;

^10^ Department of Geography, Tourism and Territorial Planning, Faculty of Geography, Tourism and Sport, University of Oradea, University Street, no. 1, Romania;

^11^ Centre for Research on Settlements and Urbanism, Faculty of Geography, Babeş-Bolyai University, Cluj-Napoca 400006; Romania;

^12^ National Institute for Economic Research Costin C. Kiriţescu, Romanian Academy, 050711Bucharest, Romania;

**^1^**^3^ Faculty of Geography, Babeş-Bolyai University, Cluj-Napoca 400006, Romania;

*corresponding. Daniel Peptenatu; Tel.: +40723683661 E-mail: peptenatu@yahoo.fr

Supplementary Material

**Table S1.** Particle analysis and fractal analysis of the treecover (T), loss (L) and cumulatively loss (CL) areas between 2000 and 2014.

| **Years** | **Type** | **Total Area [ha]** | **Count ^*^ [pixel]** | **Average Size [pixel]** | ***LCFD*** | $\boldsymbol{\Lambda}_{\mathbf{T-o-W}}$ | ***FFI*** |
| --- | --- | --- | --- | --- | --- | --- | --- |
| 2000 | T | 794,005.1 | 7,972 | 99.60 | 1.890 | 0.186 | 0.182 |
| 2001 | T | 790,775.9 | 7,987 | 99.01 | 1.889 | 0.151 | 0.174 |
|  | L | 3,229.167 | 2,980 | 1.08 | 0.419 | 1.301 | 0.002 |
| 2002 | T | 790,351.5 | 7,990 | 98.92 | 1.889 | 0.167 | 0.174 |
|  | L | 424.413 | 368 | 1.15 | 0.385 | 1.051 | 0.001 |
|  | CL | 3,653.58 | 3,228 | 1.13 | 0.441 | 1.017 | 0.002 |
| 2003 | T | 787,594.1 | 8,014 | 98.28 | 1.888 | 0.148 | 0.171 |
|  | L | 2,757.404 | 1,500 | 1.84 | 0.728 | 1.337 | 0.006 |
|  | CL | 6,410.984 | 4,383 | 1.46 | 0.622 | 0.997 | 0.004 |
| 2004 | T | 786,218.5 | 8,022 | 98.01 | 1.888 | 0.152 | 0.169 |
|  | D | 1,375.615 | 1,222 | 1.13 | 0.449 | 1.001 | 0.004 |
|  | CL | 7,786.599 | 5,265 | 1.48 | 0.633 | 0.920 | 0.004 |
| 2005 | T | 784,771.8 | 8,038 | 97.63 | 1.887 | 0.141 | 0.167 |
|  | D | 1,446.702 | 1,061 | 1.36 | 0.547 | 1.082 | 0.004 |
|  | CL | 9,233.3 | 5,859 | 1.58 | 0.678 | 0.868 | 0.005 |
| 2006 | T | 783,828.2 | 8,051 | 97.36 | 1.887 | 0.142 | 0.166 |
|  | D | 943.5825 | 719 | 1.31 | 0.495 | 1.139 | 0.004 |
|  | CL | 10,176.88 | 6,215 | 1.64 | 0.700 | 0.863 | 0.006 |
| 2007 | T | 776,091.3 | 8,289 | 93.63 | 1.883 | 0.175 | 0.161 |
|  | D | 7,736.836 | 2,680 | 2.89 | 1.019 | 1.185 | 0.015 |
|  | CL | 17,913.72 | 7,068 | 2.53 | 1.002 | 0.841 | 0.014 |
| 2008 | T | 774,767 | 8,310 | 93.23 | 1.882 | 0.173 | 0.159 |
|  | D | 1,324.311 | 1,138 | 1.16 | 0.426 | 1.073 | 0.003 |
|  | CL | 19,238.03 | 7,487 | 2.57 | 1.008 | 0.791 | 0.014 |
| 2009 | T | 773,333.9 | 8,376 | 92.33 | 1.881 | 0.171 | 0.158 |
|  | L | 1,433.138 | 1,111 | 1.29 | 0.506 | 1.172 | 0.003 |
|  | CL | 20,671.17 | 7,787 | 2.65 | 1.030 | 0.769 | 0.015 |
| 2010 | T | 771,235.1 | 8,439 | 91.39 | 1.881 | 0.164 | 0.157 |
|  | L | 2,098.804 | 1,571 | 1.34 | 0.534 | 1.161 | 0.004 |
|  | CL | 22,769.97 | 8,291 | 2.75 | 1.057 | 0.784 | 0.016 |
| 2011 | T | 770,087.4 | 8,479 | 90.82 | 1.880 | 0.170 | 0.156 |
|  | L | 1,147.661 | 685 | 1.68 | 0.699 | 1.112 | 0.01 |
|  | CL | 23,917.63 | 8,495 | 2.82 | 1.075 | 0.772 | 0.018 |
| 2012 | T | 765,951.8 | 8,635 | 88.70 | 1.878 | 0.161 | 0.153 |
|  | L | 4,135.585 | 2,534 | 1.63 | 0.707 | 1.116 | 0.008 |
|  | CL | 28,053.22 | 9,140 | 3.07 | 1.140 | 0.753 | 0.021 |
| 2013 | T | 764,927.5 | 8,663 | 88.30 | 1.878 | 0.138 | 0.152 |
|  | L | 1,024.328 | 1,185 | 0.86 | 0.312 | 1.152 | 0.001 |
|  | CL | 29,077.55 | 9,608 | 3.03 | 1.135 | 0.725 | 0.021 |
| 2014 | T | 764,001.9 | 8,699 | 87.83 | 1.877 | 0.167 | 0.151 |
|  | L | 925.572 | 1,017 | 0.91 | 0.309 | 1.119 | 0.001 |
|  | CL | 30,003.12 | 9,951 | 3.02 | 1.134 | 0.733 | 0.021 |
|  | CG | 10,508.67 | 9,215 | 1.14 | 0.576 | 0.671 | 0.003 |

T = treecover areas; L = loss areas; CL = cumulatively loss areas and CG = cumulatively gain areas; *Count = number of particles (forest patches).

Except for the number of particles (increment trend from 2000 to 2014), all indicators associated with treecover areas are decreased from 2000 to 2014 because as cumulative loss areas increase, forest patches generate a decrease in LCFD (decreases treecover connectivity), and FFI (increase forest degradation).$\Lambda_{T-o-W}$ has increases or decreases in this period imposed by the deforestation pattern.

**Table S2.** Spearman correlation between particles and fractal analysis parameters.

| **Loss areas** | | | | | | |
| --- | --- | --- | --- | --- | --- | --- |
| Parameter | Count | Total Area | Average Size | *LCFD* | *FFI* | $\boldsymbol{\Lambda}_{\mathbf{T-o-W}}$ |
| Count | 1 | 0.86 | 0.16 | 0.34 | -0.19 | -0.03 |
| Total Area | 0.86 | 1 | 0.56 | 0.71 | -0.03 | 0.03 |
| Average Size | 0.16 | 0.56 | 1 | 0.96 | 0.19 | 0.07 |
| *LCFD* | 0.34 | 0.71 | 0.96 | 1 | 0.21 | 0.10 |
| *FFI* | −0.19 | −0.03 | 0.19 | 0.21 | 1 | 0.24 |
| $\Lambda_{T-o-W}$ | −0.03 | 0.03 | 0.07 | 0.10 | 0.24 | 1.00 |
| **Cumulative loss areas** | | | | | | |
| Parameter | Count | Total Area | Average Size | *LCFD* | *FFI* | $\Lambda_{T-o-W}$ |
| Count | 1 | 0.94 | 0.82 | 0.82 | 0.85 | −0.09 |
| Total Area | 0.94 | 1 | 0.96 | 0.96 | 0.97 | 0.03 |
| Average Size | 0.82 | 0.96 | 1 | 1 | 1 | 0.08 |
| *LCFD* | 0.82 | 0.96 | 1 | 1 | 1 | 0.08 |
| *FFI* | 0.85 | 0.97 | 1 | 1 | 1 | 0.05 |
| $\Lambda_{T-o-W}$ | −0.09 | 0.03 | 0.08 | 0.08 | 0.05 | 1 |
| **Treecover areas** | | | | | | |
| Parameter | Count | Total Area | Average Size | *LCFD* | *FFI* | $\Lambda_{T-o-W}$ |
| Count | 1 | −1 | −1 | −1 | −1 | 0.25 |
| Total Area | −1 | 1 | 1 | 1 | 1 | −0.25 |
| Average Size | −1 | 1 | 1 | 1 | 1 | −0.25 |
| *LCFD* | −1 | 1 | 1 | 1 | 1 | −0.25 |
| *FFI* | −1 | 1 | 1 | 1 | 1 | −0.25 |
| $\Lambda_{T-o-W}$ | 0.25 | −0.25 | −0.25 | −0.25 | −0.25 | 1 |


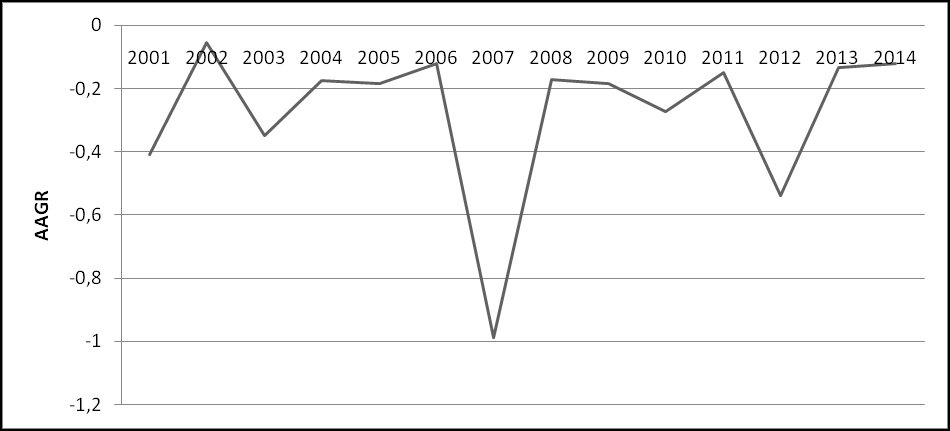


**Figure S1.** Average annual growth rate of total forest in the Apuseni Mountains between 2001 and 2014. It highlights the dynamics of the reduction of treecover, depending on the loss areas.
